# Effect of drying methods on *Acetobacter xylinum* bacterial cellulose aerogels and cryogels

**DOI:** 10.1038/s41598-026-42244-1

**Published:** 2026-03-05

**Authors:** Şebnem Sözcü, Jakub Wiener, Jaroslava Frajová, Mohanapriya Venkataraman, Blanka Tomková, József Kalmár, Attila Forgács, Jiří Militký

**Affiliations:** 1 Department of Material Engineering, Faculty of Textile Engineering, Technical University of Liberec, 46117 Liberec, Czech Republic; 2 HUN-REN-DE Mechanisms of Complex Homogeneous and Heterogeneous Chemical Reactions Research Group, Department of Inorganic and Analytical Chemistry, University of Debrecen, Egyetem tér 1, Debrecen, H-4032 Hungary

**Keywords:** *Acetobacter xylinum*, Lyophilization, Supercritical CO_2_, nanoporous bacterial cellulose, aerogels, and cryogels, Biotechnology, Chemistry, Engineering, Materials science

## Abstract

Bacterial cellulose (BC) pellicles were produced from *Acetobacter xylinum *using a simple, additive-free, and low-cost static cultivation method consistent with sustainable and green bioprocessing principles. Two post-synthesis drying routes were compared: supercritical carbon dioxide (scCO_2_) drying following acetone solvent exchange and direct lyophilization without chemical additives or pre-freezing. The resulting BC aerogels and cryogels were characterized by SEM, confocal microscopy, BET analysis, FTIR spectroscopy, EDS, and geometrical evaluation with a particular emphasis on nanostructure, porosity, and network integrity. scCO_2_-dried BC aerogels exhibited a well-preserved three-dimensional nanofibrillar network, achieving a BET surface area (123 m^2^/g), large pore volume (0.36 cm^3^/g), and an average pore diameter of 10 nm. Confocal microscopy revealed higher surface roughness (Rz up to ~ 58 μm), reflecting a more developed and heterogeneous surface topography. Lyophilized BC cryogels showed lower surface area (51 m^2^/g) and pore volume (0.13 cm^3^/g); however, SEM and confocal analyses indicated that the nanofibrillar network and three-dimensional architecture were largely retained, with only localized fibril aggregation and reduced roughness (~ 28–30 μm). EDS confirmed high chemical purity in scCO_2_-dried aerogels, while minor inorganic traces detected in cryogels were attributed to residual components from the tea-based culture medium. Although scCO_2_ drying provided slightly superior structural preservation and textural properties, the porous architecture remained comparable between the two methods. Overall, additive-free BC pellicles produced by static cultivation and processed via limited pre-freezing followed by lyophilization provided a structurally comparable and more sustainable alternative, offering a practical balance between textural performance and processing simplicity. These findings underscore the potential of simplified drying strategies for the sustainable fabrication of BC-based porous materials without compromising structural functionality.     .

## Introduction

*Acetobacter xylinum* is one of the microbial strains that produces bacterial cellulose (BC). In contrast to cellulose obtained from plants, BC has a highly pure nanofibrillar network that is distinguished by exceptional crystallinity, tensile strength, and biocompatibility. It is also free of pectins, xylan, lignin, and hemicelluloses. Because of these inherent qualities, BC is a desirable option for use in advanced functional materials, packaging, environmental remediation, and biomedical engineering^1–3^. Because the procedure is easy to use, economical, and produces consistently high-quality pellicles, the sustainable production of BC using static cultivation is still very pertinent in laboratory settings.

Drying after synthesis is essential for maintaining the structural integrity and functional characteristics of BC aerogels (BCAs). Performance is limited in applications that demand large surface area and porosity because conventional drying techniques, such as oven or air drying usually result in substantial pore collapse and dense structures^4^.

However, by reducing capillary stress during water removal, advanced methods such as supercritical carbon dioxide (scCO_2_) drying and freeze-drying (lyophilization) allow for the preservation of the three-dimensional porous architecture. In order to guarantee regulated sublimation of ice crystals, lyophilization typically necessitates a pre-freezing phase; however, simplified protocols without pre-freezing have also been investigated, offering reduced processing complexity^5,6^. scCO_2_ drying, on the other hand, is thought to be very successful in maintaining network morphology and creating low-density aerogels. It entails solvent exchange with CO_2_-miscible organic solvents like acetone or ethanol^4,7,8^. scCO_2_ drying frequently produces materials with lower bulk density, larger porosity, and greater surface area than those obtained from freeze-drying, according to comparative investigations on cellulose-based aerogels^4^. Nevertheless, cryogels may display advantageous mechanical and textural properties depending on drying procedures and freezing circumstances^5,6^. However, there are still few direct comparisons between BC aerogels made by lyophilization without pre-freezing and scCO_2_ drying, despite these revelations.

Recent developments in cellulose-based materials have shown how adaptable nanostructured cellulose is in a variety of functional applications, such as ecologically relevant systems, porous solids, and sustainable nanomaterials. For instance, current research has emphasized the creation of nanoparticles generated from cellulose with customized surface chemistry and porosity for enhanced material design^9,10^. In the context of environmental technologies and sustainable energy systems, where structure-property correlations are crucial, cellulose-based porous designs have also been investigated^11,12^.

Simultaneously, attempts to enhance the scalability and sustainability of cellulose processing have highlighted the significance of green fabrication methods that preserve structural integrity without requiring complicated chemical modification^13,14^. In this regard, the current study compares drying methods for bacterial cellulose made without the use of additives, with a focus on the effects of processing variables on nanofibrillar architecture and porosity.

In this study, two different drying procedures were applied to BC pellicles produced by static cultivation: (i) lyophilization without the traditional pre-freezing phase, and (ii) supercritical CO_2_ (scCO_2_) drying following solvent exchange from water to acetone. The resulting BC aerogels and cryogels were thoroughly characterized using scanning electron microscopy (SEM), confocal microscopy, energy-dispersive X-ray spectroscopy (EDS), Brunauer–Emmett–Teller (BET) surface area analysis/nitrogen sorption, Fourier-transform infrared spectroscopy (FTIR), and geometrical evaluation. This study compares two drying methods and examines how they affect the microstructural and surface properties of BC membranes synthesized without additives.

## Materials and methods

### Materials

The Czech Collection of Microorganisms, Department of Experimental Biology (Brno, Czech Republic), provided the *Acetobacter xylinum* (CCM 2360) strain. Sugar (TTD company – crystal white sugar) was purchased from a local market in Liberec, Czech Republic, and used as the carbon source for fermentation, while black tea (PG Tips Loose Leaf Finest Catering Black Tea) was used as the nitrogen source. To purify the BC pellicles, sodium carbonate (Na_2_CO_3_; molecular weight: 105.99), anhydrous, was purchased from Chemapol (Prague, Czech Republic), as described in the reference article^15^. All experiments were performed using deionized water. Acetone (Merck, ACS reagent grade) was used for solvent exchange. The purification procedure applied in this study was designed to remove residual culture medium and bacterial remnants to a level sufficient for structural and physicochemical characterization. The present work does not address biomedical or clinical applications, for which more aggressive alkaline treatments (e.g., NaOH-based protocols) are commonly employed to meet medical-grade purity requirements. The selected washing conditions were optimized to preserve the native fibrillar architecture of BC while ensuring adequate removal of soluble impurities.

Since all synthesis and purification steps were consistent across samples, the bacterial cellulose pellicle preparation followed the protocol described in Article^15^, with the exception that no fabric support was used for stabilization in this experiment. The drying methods varied: Samples 1 and 2 were dried using supercritical CO_2_, while Samples 3, 4, and 5 underwent lyophilization. A total of five samples were prepared for analysis. Morphological and chemical characterization, including scanning electron microscopy (SEM), Fourier-transform infrared spectroscopy (FTIR), and confocal microscopy, focused on Samples 1, 2, 3, and 5, which were selected to represent the range of sample thicknesses, with Sample 3 and Sample 5 corresponding to the thinnest and thickest specimens, respectively. Energy-dispersive X-ray spectroscopy (EDS) was conducted on representative samples from each drying method to compare elemental composition. Brunauer–Emmett–Teller (BET) surface area measurements and geometrical analyses were performed on all samples to provide a comprehensive assessment of porosity.

### Drying procedures

#### Lyophilization

Purified wet BC pellicles were lyophilized using a fully automatic Leosmak LEO-004 freeze dryer (Wroclaw, Poland). Instead of employing a separate or complex freezing step, the samples were pre-frozen directly in the lyophilizer for 3 h at − 30 °C using cooled shelves, ensuring process continuity during lyophilization. This freeze-drying approach yields highly porous three-dimensional polymeric networks known as cryogels. In this work, the term *cryogel *is therefore used to describe the materials obtained via lyophilization. 

The drying process was subsequently carried out for 48 h at − 33 °C under a vacuum pressure of 36 Pa, with mild shelf heating (25–30 °C). Although lyophilization is often described as being performed without a distinct pre-freezing stage, a brief and integrated pre-freezing period was applied here as part of the drying process rather than as an independent step. Longer pre-freezing durations were intentionally avoided, as the authors have previously shown that extended freezing can induce surface crystallization and pore blockage in BC aerogels, negatively affecting pore structure and accessibility^15^.

#### Supercritical CO_2_ Drying

Water in the large BC pellicle samples was gradually exchanged with acetone to obtain a solvent suitable for supercritical drying. To guarantee full replacement, the water was first exchanged with a water–acetone (1:1) mixture, and then with pure acetone. Acetone was replaced with fresh acetone every 3 days, for a total of three cycles, to remove traces of water from the solvent. Solvent exchange was thus conducted in multiple stages with periodic renewal of fresh acetone to approach equilibrium conditions; the exchange schedule was selected with consideration of sample thickness to ensure effective solvent replacement prior to supercritical drying. Following solvent exchange, the acetone-saturated samples were dried for 48 h at 65 °C and 90 bar in a supercritical CO_2_ dryer, resulting in aerogels with retained porous architecture. The drying process and the equipment are described in a previously published paper^16^. Among the solvents tested (ethanol, methanol, acetone), acetone yielded the most stable specimens with minimal shrinkage and consistent results, confirming its suitability for further use.

### Characterization methods

Morphological Analysis: The morphological structure of BC was analyzed using a Zeiss Ultra Plus scanning electron microscope (SEM, Potsdam, Germany) operated at an accelerating voltage of 2 kV. Samples were coated with an Au/Pd (80/20) alloy. Elemental distribution and quantitative analysis were performed using an SEM-attached energy-dispersive X-ray spectrometer (EDS) to determine the elemental composition. The Nicolet iS50 FTIR spectrometer (Madison, WI, USA) was used to conduct Fourier-transform infrared spectroscopy (FTIR) measurements. The system operates in the 400–4000 cm^−1^ range and is controlled by the OMNIC™ 9 software package, which provides spectral libraries and enables the separation of overlapping spectral bands. The primary measuring accessory was an attenuated total reflectance (ATR) attachment, allowing measurements in the spectral range of 100–4000 cm^−1^.

Geometrical Analysis: Bulk density and porosity were calculated from the measured mass, volume, and thickness, following methodologies described in previous studies^15,17^. Furthermore, the water content of bacterial cellulose samples was evaluated gravimetrically using the difference between wet and dry mass, which was computed as the percentage mass loss during drying $${\raise0.7ex\hbox{${W_{{wet}} - W_{{dry}} }$} \!\mathord{\left/ {\vphantom {{W_{{wet}} - W_{{dry}} } {W_{{wet}} }}}\right.\kern-\nulldelimiterspace} \!\lower0.7ex\hbox{${W_{{wet}} }$}} \times 100$$.

3D Surface Roughness and Topography Analysis: Feature size and configuration significantly impact surface quality, functionality, and final product performance. Measuring surface roughness is crucial for achieving high performance standards in finished products^18^. Confocal microscopy is a practical way to obtain three-dimensional descriptions of objects. A novel technique takes advantage of confocal microscopy’s ability to quantify rough surfaces. The microscope optically sections the surface, and a computer converts the sections into digital images and a topographic map. The computer analyzes the topographic map using a standard technique to generate a roughness parameter that describes the texture of the surface^19^. The profile is obtained from the primary profile by suppressing the long wave component using a high-pass filter with a cutoff value of λ_c_. The roughness profile is used to calculate the surface texture parameter (R-parameter)^18,20^. Rz is the maximum height of the roughness profile and indicates line roughness. The maximum peak height Zp and the maximum valley depth Zv are the highest and lowest points within the reference length. The difference is the maximum height roughness Rz (= Zp-Zv). Rz is also commonly used for quality control^18,21^.

In this study, the surface roughness of the bacterial cellulose specimens was analyzed using a confocal scanning infrared (IR) laser microscope (Olympus Corporation, Hachioji-shi, Tokyo, Japan). Measurements were performed with LEXT-OLS3100 software MM6-ASPS, equipped with a 405 nm laser and 20× objective lenses, in accordance with the ČSN EN ISO 21920-2 (014450) standard^22^. Statistical analysis was performed using analysis of variance (ANOVA), followed by Tukey’s post-hoc test for multiple comparisons.

Specific Surface Area and Pore Volume Analysis: The BET (Brunauer–Emmett–Teller) equation was used to calculate the specific surface area and pore size distribution of the bacterial cellulose aerogels and cryogels based on nitrogen adsorption–desorption isotherms obtained at 77 K using an Autosorb 6100 FKM XR-XR analyzer (Anton Paar) and Kaomi Autosorb software. The samples were degassed at 50 °C for 48 h before measurement. The BET analysis was performed within the linear relative pressure range of 0.05–0.3 (p/p₀).

## Results and discussion

### Surface morphology and microstructure analysis 

**Fig. 1:**
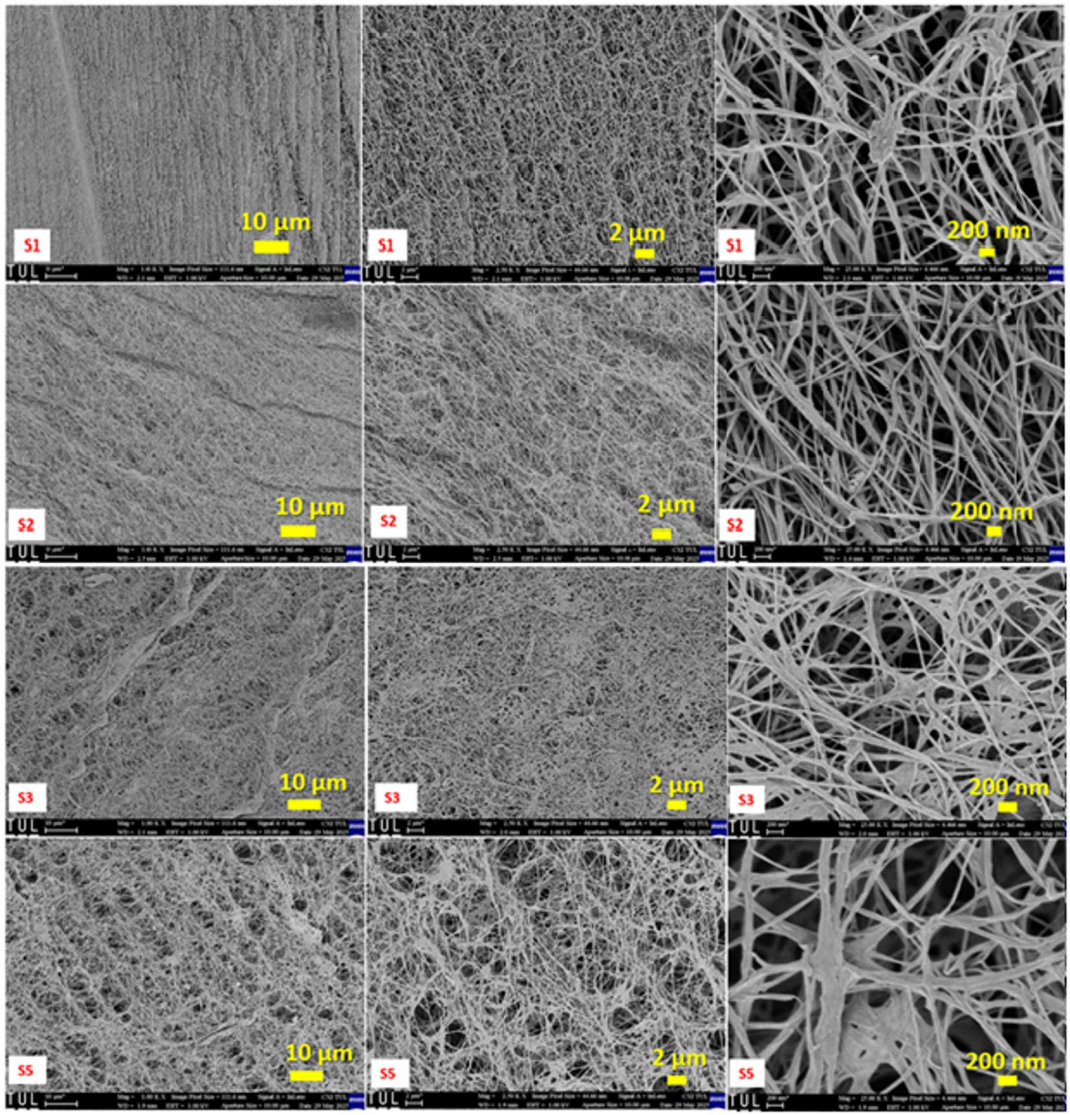
Comparison of SEM image analysis of BC aerogels and cryogels.

As seen in Fig. 1, the SEM micrographs present a thorough comparison between cryogels produced by lyophilization with minimum freezing (samples 3–5) and bacterial cellulose (BC) aerogels dried using supercritical CO_2_ (scCO_2_) after acetone solvent exchange (samples 1–2). To clarify the distinction between individual specimens discussed in this section, Table 1 summarizes the standardized sample identifiers, thickness values, and corresponding drying conditions, which account for the observed morphological variations between samples 1 and 2 as well as samples 3 and 5.SEM micrographs were acquired at three different magnifications (1000×, 2500×, and 25,000×), corresponding to scale bars of 10 μm, 2 μm, and 200 nm, respectively., allowing for the evaluation of nanoscale fibrillar organization and microscale porosity.

By eliminating water before drying by acetone solvent exchange, the scCO_2_ drying technique reduces capillary stress and eliminates liquid–gas surface tension during CO_2_ venting. The delicate three-dimensional nanofibrillar architecture of BC is successfully preserved by this procedure. scCO_2_-dried aerogels (samples 1–2) show a uniform and highly porous shape at low magnification (1000× ) with no signs of surface densification or large-scale collapse. Images with an intermediate magnification of 2500× verify consistent network connectivity and homogenous pore distribution. In sample S2, localized features distinct from the fibrillar network can be observed, which are consistent with minor residual components also indicated by EDS analysis. These features are likely associated with trace medium residues remaining after purification. The washing condition applied in this study was optimized to preserve the native fibrillar architecture rather than to achieve medical-grade purity, which may account for the presence of limited residual species without affecting the overall structural integrity of the aerogel network.

Individual cellulose fibrils, which are usually 45–55 nm in diameter, look smooth, well-separated, and isotropically distributed at higher magnifications (200 nm, 25,000×), forming an open, sponge-like network. Effective solvent exchange and maintenance of the original BC network are confirmed by the well-dispersed and linked pores, which show little structural distortion after drying. The aerogels thus display the high porosity, low density, and nanoscale architecture typical of cellulose aerogels that are structurally intact.

On the other hand, cryogel samples (3–5) that underwent direct lyophilization with a brief integrated pre-freezing time of 3 h show greater microstructural variation while maintaining the overall nanofibrillar structure. Cryogels exhibit irregular porosity with areas of localized densification at 1000× magnification. Sample 3 shows localized fiber bundling and thickening at fibril junctions, although the fibrous network is still largely open. Smaller pores appear somewhat constricted rather than totally shut at higher magnifications, suggesting localized compaction as opposed to overall network collapse. Sample 5 shows less pore openness and more noticeable densification, with fibrils combining into compact, sheet-like areas. In general, cryogels have uneven pore distribution and heterogeneous density. These characteristics are in line with tensions caused by sublimation that result from limited ice templating during the brief pre-freezing phase.

Importantly, SEM observations indicate that the short, continuous pre-freezing duration applied here did not result in severe pore blockage or complete structural degradation, as the three-dimensional nanofibrillar network remains largely preserved across all cryogel samples. This behavior differs from the effects of extended or conventional pre-freezing reported in the authors’ previous study^15^ where longer freezing times promoted surface crystallization, pore obstruction, and lamination. In that work, rapid liquid-nitrogen (LN_2_) pre-freezing yielded highly porous, interwoven networks, whereas conventional freezing produced denser, compact morphologies. The present scCO_2_-dried aerogels (samples 1–2) closely resemble the open, uniform nanofiber networks observed for LN_2_-pre-frozen BC aerogels, while the cryogels (samples 3–5) exhibit compaction behavior similar to conventionally frozen BC materials.

Taken together, both studies^15^ confirm that processing conditions—particularly drying strategy and freezing dynamics—play a decisive role in preserving the nanostructural integrity of bacterial cellulose. Rapid fluid replacement (acetone exchange followed by scCO_2_ drying) or rapid freezing approaches favor isotropic, highly porous networks, whereas slower or incomplete phase transitions, such as lyophilization without deep or prolonged freezing, lead to localized densification and reduced pore connectivity. The present SEM results therefore demonstrate that while scCO_2_ drying provides superior structural uniformity, additive-free lyophilization with limited pre-freezing can still preserve the essential nanofibrillar architecture, albeit with increased heterogeneity, supporting its viability as a simpler and more sustainable processing route.

**Fig. 2:**
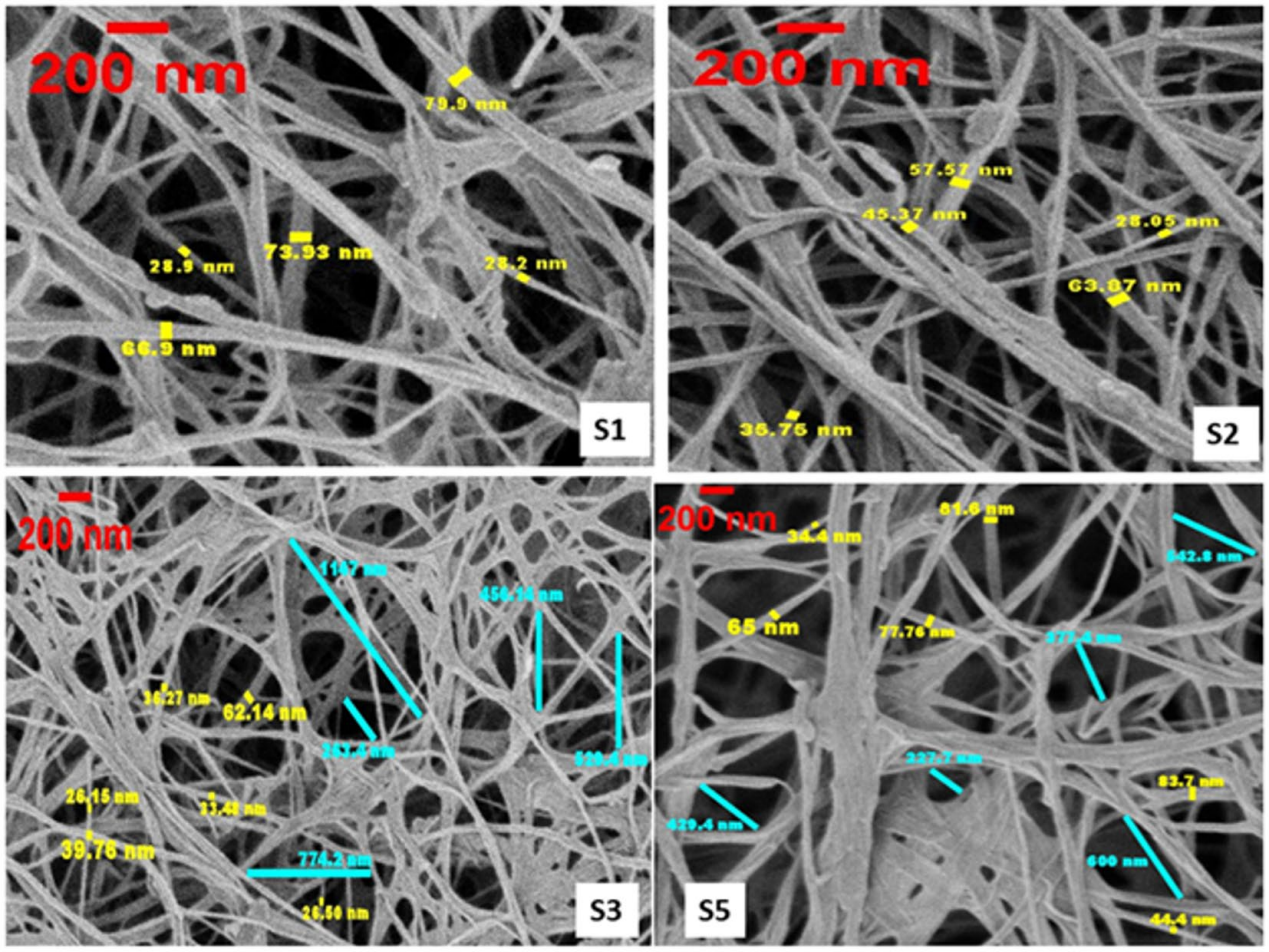
Fiber diameter of BC Aerogels and Cryogels.

The quantitative SEM analysis of fiber diameters and inter-fibril distances performed using ImageJ software revealed clear distinctions between the drying routes used for aerogels (samples 1–2) and cryogels (samples 3–5), as shown in Fig. 2. The aerogel samples (S1 and S2) exhibited uniformly distributed nanofibers with average diameters of 55.58 nm and 46.12 nm, respectively, forming a dense yet interconnected fibrillar network. This fine and homogeneous morphology corresponds well with the high BET surface area (123 m^2^/g) and large pore volume (0.36 cm^3^/g) obtained for these samples, confirming that acetone solvent exchange and scCO_2_ drying effectively preserved the native mesoporous structure while minimizing fibril aggregation.

In contrast, the cryogel samples (S3 and S5) demonstrated more heterogeneous morphologies. S3 showed the thinnest fibers (average: 37.38 nm) but the largest inter-fibril distance (average: 634.24 nm), suggesting partial structural collapse and the formation of wide, irregular voids due to sublimation-induced stress. Conversely, S5 exhibited thicker fibers (average: 64.50 nm) and a smaller mean spacing (average: 435.48 nm), indicative of localized densification and fibril fusion. These structural deviations are consistent with their lower BET surface areas (≈ 51 m^2^/g) and reduced pore volumes (0.13 cm^3^/g), confirming a loss of mesoporosity relative to scCO_2_-dried aerogels. Overall, the quantitative morphological analysis confirms that scCO_2_ drying produces a more uniform, mesoporous, and dimensionally stable nanofibrillar architecture, whereas lyophilization without prior deep freezing yields partially collapsed cryogels with uneven pore structures and reduced nanoscale definition, in agreement with BET and SEM observations.

### Elemental distribution and quantitative analysis

**Fig. 3:**
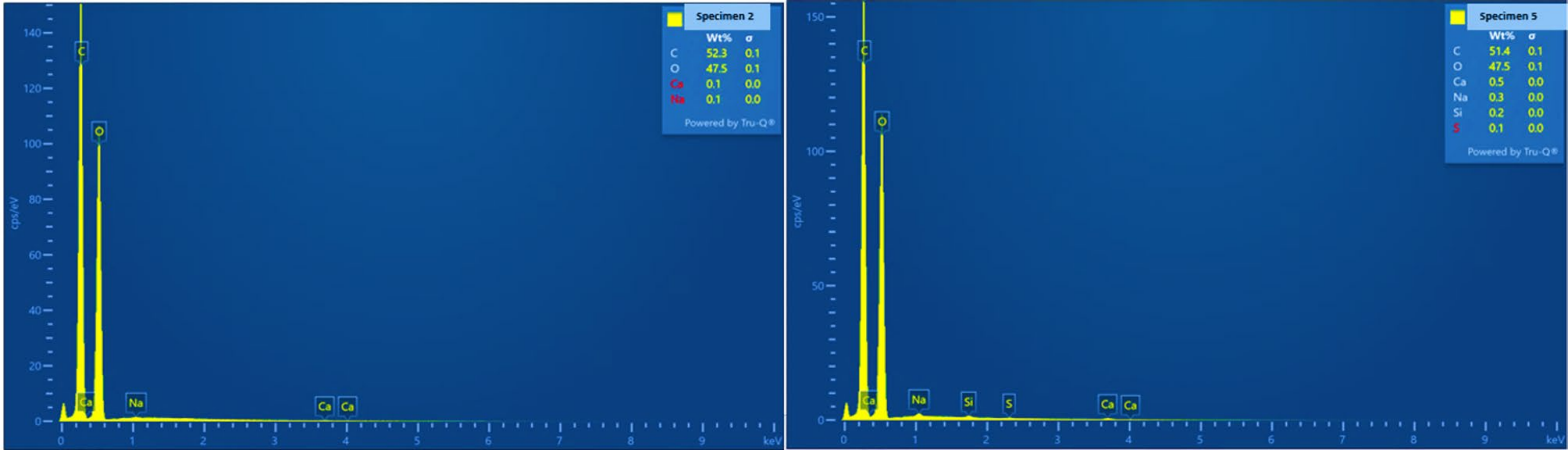
EDS results of Sample 2 and 5 Bacterial Cellulose Aerogels and Cryogels.

Figure 3 shows the EDS spectra of bacterial cellulose (BC) pellicles, revealing compositional changes between the aerogel (Sample 2) and the cryogel (Sample 5). Both spectra are characterized by prominent carbon (C) and oxygen (O) peaks, which are typical of the polysaccharide structure of bacterial cellulose. Sample 2, dried with supercritical CO_2_ following acetone solvent exchange, is mostly composed of carbon (52.3 wt%) and oxygen (47.5 wt%), with trace amounts of calcium (0.1 wt%) and sodium. The nearly stoichiometric C: O ratio indicates a highly pure cellulose matrix, suggesting that acetone exchange and scCO_2_ drying efficiently retained the polymeric structure and prevented elemental contamination. Multiple studies have demonstrated greater textural retention (larger surface area and porosity, reduced shrinkage) when scCO_2_ drying is employed on cellulose gels, emphasizing the importance of solvent exchange quality for the ultimate purity and structure^23,24^.

The cryogel (Sample 5), produced by direct freezing during lyophilization for three hours without prior solvent exchange, has a comparable C: O ratio (C = 51.4 wt%, O = 47.5 wt%) but contains slightly higher traces of minor elements, including calcium (0.5 wt%), sodium (0.3 wt%), silicon (0.2 wt%), and sulfur (0.1 wt%). These traces are likely caused by naturally occurring minerals in tea, as well as minimal pickup from labware, sample holders, and handling, rather than from additional salts in the medium. Black tea leaves and infusions contain significant amounts of Ca, Na, S, and other minerals, which can remain at extremely low levels on biopolymer surfaces after washing and drying^25^. The formation of these peaks can also be attributed to localized mineral particle deposition on the cryogel surface, which may become more concentrated as water sublimes during lyophilization.

The observed compositional fluctuations are consistent with the morphological patterns exhibited in the SEM micrographs. The aerogel sample showed an open, homogeneous nanofibrillar structure with low levels of contaminants, indicating a clean elemental profile. In contrast, the cryogel sample exhibited a denser, partially collapsed morphology with thicker fiber bundles, which was consistent with the presence of small inorganic residues. These findings indicate that the lyophilization process, while simpler, may allow ambient or surface-associated components to become embedded in the compacted fibrillar matrix.

The EDS analysis confirmed that both aerogels and cryogels are chemically dominated by cellulose. However, the aerogel sample exhibited superior compositional purity and structural preservation, whereas the cryogel sample contained trace external mineral residues and partial morphological compaction. This result demonstrates that scCO_2_ drying after solvent exchange produces cleaner and more stable bacterial cellulose aerogels, while lyophilization without solvent mediation can introduce minor extrinsic elemental residues during processing.

### Geometrical analysis

Table 1 displays the structural and physical properties of five samples subjected to lyophilization and supercritical CO_2_ (scCO_2_) drying. The measured parameters include porosity, dry mass, bulk density, thickness, mass, and sample dimensions, both before and after drying (all parameters were measured at five different locations for each sample due to surface unevenness). These parameters are essential for evaluating the effects of the drying techniques on density, porosity, and structural integrity. The geometrical parameters of the wet BC pellicles are presented as baseline reference values to quantify dimensional and volumetric changes induced by the respective drying methods. These wet-state measurements are not intended as a performance comparison between samples but serve to establish the initial hydrated geometry from which shrinkage and density variations were determined.

**Table 1:** Geometrical analysis of BC samples dried with sc CO_2_ and lyophilization.

Sample no	Drying method	Before dried			After dried				
		Wet thickness (cm)	Wet mass (g)	Dimension (cm)	Dry thickness (cm)	Dry Mass (g)	Bulk density (mg/cm^3^)	Porosity (%)	Dimension (cm)
S1 (mean)	sc CO_2_	0.68	76.56	9 × 9	0.55	0.61	14	99.1	8.8 × 9.2
S2 (mean)		0.95	103.83	9.5 × 9.5	0.78	0.87	13	99.2	9.3 × 9.3
Total Mean		0.81	90.19	–	0.66	0.74	13.5	99.15	–
Standard deviation		0.19	19.28	–	0.16	0.18	0.70	0.07	–
S3 (mean)	Lyophilization	0.28	31.44	10 × 10	0.13	0.34	28	98.18	9 × 10
S4 (mean)		0.65	78.38	10 × 10	0.57	0.73	13	99.14	9.6 × 9.8
S5 (mean)		0.81	181.5	13.5 × 14.2	0.67	1.42	12	99.22	12.9 × 13.5
Total mean		0.58	97.10	–	0.45	0.83	17	98.84	–
Standard deviation		0.27	76.76	–	0.28	0.54	8.96	0.57	–

Bacterial cellulose (BC) pellicles dried by lyophilization and supercritical CO_2_ drying with acetone solvent exchange exhibit clear structural and physical variations in their properties. The wet samples varied slightly in thickness and mass prior to drying; aerogels had an average wet thickness of 0.81 ± 0.19 cm and a wet mass of 90.19 ± 19.28 g, whereas cryogel samples had an average wet thickness of 0.58 ± 0.27 cm and a wet mass of 97.10 ± 76.76 g. The greater variability observed in cryogels points to either uneven water content prior to drying or less consistent gel formation. The gravimetrically determined water content exceeded 99% for both aerogels and cryogels based on the wet and dry mass values shown in Table 1, indicating that the BC pellicles were highly hydrated before drying.

Both drying methods produced extremely lightweight, highly porous bacterial cellulose (BC) aerogels and cryogels with only marginal differences in geometry and density. The aerogel samples showed an average density of 13.5 ± 0.70 mg/cm^3^ and a porosity of 99.15 ± 0.07%, while the cryogel samples had a slightly higher density of 17 ± 8.96. mg/cm^3^ and a porosity of 98.84 ± 0.57%. The comparably high porosity and water content data show that neither drying method significantly collapsed the hydrated BC network at the macroscopic level. These results confirm that both techniques effectively preserved the intrinsic open-pore structure of BC, yielding materials with over 98% porosity. The thickness reduction after supercritical CO₂ drying was approximately 17–19%, while lyophilized samples exhibited shrinkage ranging from 12% to 53%. These values fall within the range reported in the literature for additive-free bacterial cellulose aerogels and cryogels, where dimensional changes are strongly influenced by gel thickness, water content, and drying conditions [^36^].

Although scCO_2_ drying provided slightly more reproducible geometrical outcomes and greater uniformity due to precise solvent exchange and controlled depressurization, the lyophilization process achieved nearly equivalent structural properties. Considering its simpler operation, lower equipment cost, and scalability, lyophilization can therefore be regarded as a viable and more accessible alternative for producing BC aerogels with comparable bulk characteristics. The primary advantages of scCO_2_ drying lie in morphological refinement and reproducibility, while lyophilization remains a practical, energy-efficient method for sustainable large-scale fabrication when small deviations in microstructure are acceptable.

The outcomes of this study’s lyophilization are largely in line with those of our earlier investigation^15^. Here, the cryogels showed low density (12–28 mg/cm^3^) and very high porosity (98.18–99.22%), demonstrating that freeze-drying successfully preserves the three-dimensional nanofiber network. However, there was greater variation in density and thickness, which was consistent with earlier research^15^, in which variations in freezing conditions and structural support had a major impact on aerogel uniformity. Minor pore collapse and dimensional shrinkage were likely caused by the lack of pre-freezing control or supporting frameworks in this investigation, which is in line with the documented sensitivity of BC structure to freezing rate and ice crystal formation. Overall, both experiments show that, whereas lyophilization consistently yields lightweight, porous BC cryogels, optimized freezing and preparation conditions have a significant impact on the structural consistency of these aerogels.

### Functional group and chemical structure analysis

**Fig. 4:**
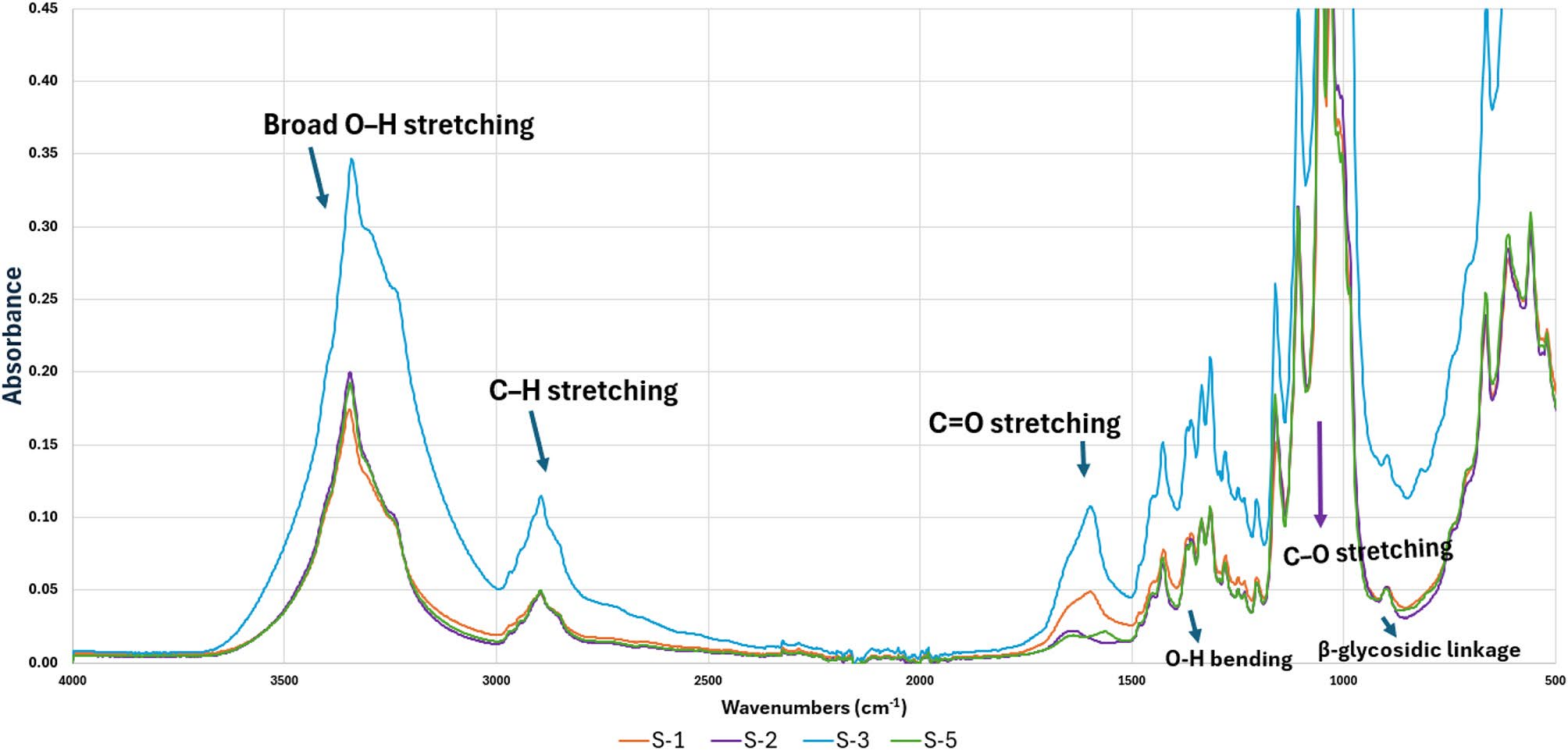
FT-IR results of bacterial cellulose aerogels and cryogels.

The FTIR spectra (Fig. 4) of samples S1, S2, S3, and S5 reveal the characteristic vibrational bands of cellulose I, indicating that the basic chemical structure of bacterial cellulose remained intact despite both supercritical CO_2_ drying and lyophilization. The broad O–H stretching band at ~ 3340 cm^−1^ indicates intra- and intermolecular hydrogen bonding within the cellulose network, while the C–H stretching peak at ~ 2890 cm^−1^ corresponds to aliphatic –CH_2_ groups in the polysaccharide backbone. The weak absorption at 1640 cm^−1^ is attributed to O–H bending of adsorbed water, whereas the C=O stretching band near 1730–1650 cm^−1^ is associated with trace carbonyl or ester groups originating from bacterial metabolism.

C–O stretching peaks at 1160–1030 cm^−1^ and the β-glycosidic linkage at ~ 895 cm^−1^ indicate the presence of the cellulose I crystalline structure. The spectral patterns and peak intensities of S1, S2, and S5 are almost identical, indicating that the aerogel samples and one of the cryogel samples have similar hydrogen-bonded structures and moisture content. S3 exhibits a slightly broader O–H stretching band and moderately enhanced intensity in the C–O stretching region. As all samples were processed under identical freezing and lyophilization conditions, these spectral variations are attributed to differences in initial sample thickness rather than incomplete drying. The thinner geometry of S3 likely promoted faster ice front propagation and localized rearrangement of the fibrillar network during freezing, leading to subtle differences in hydrogen bonding and hydroxyl accessibility. Overall, no new functional groups or degradation signals were observed across the spectra, indicating the chemical stability of the aerogels and cryogels.

These findings are consistent with typical cellulose I FTIR band assignments reported by Hospodarová et al. (2018)^26^, Poletto et al. (2014)^27^, and Wu et al. (2014)^28^, indicating that the drying method has a greater influence on hydrogen-bond arrangement and residual hydration than on chemical composition.

### 3D surface roughness and topography analysis

Confocal scanning infrared (IR) laser microscopy enables high-resolution three-dimensional analysis of bacterial cellulose pellicle surfaces, allowing quantitative evaluation of surface roughness parameters essential for material, food, and healthcare applications. Additionally, laser-based surface texturing offers a promising approach to tailor both surface roughness and chemistry in diverse materials^15,29,30^.

**Fig. 5:**
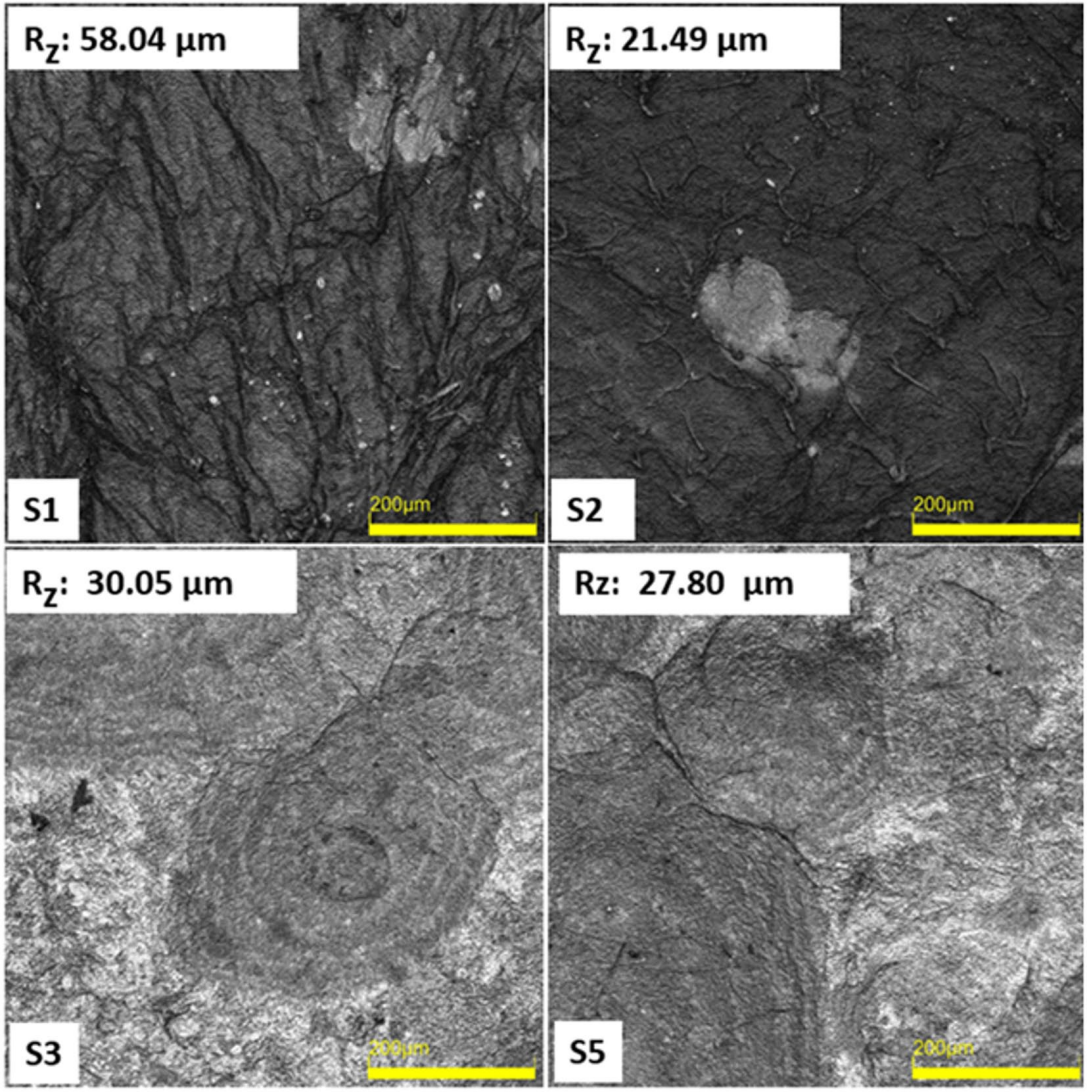
Surface roughness analysis (Objective: 20× for all images. Scale bar represents 200 μm. Rz: mean roughness depth).

CLSM (confocal laser scanning microscopy) images (Fig. 5) show distinct differences in the surface topography of bacterial cellulose (BC) pellicles prepared by supercritical CO_2_ drying with acetone solvent exchange (Samples 1–2) and lyophilization with short, direct freezing (Samples 3–5). Surface roughness (Rz) values vary significantly (Sample 1: 58.04 μm, Sample 2: 21.49 μm, Sample 3: 30.05 μm, and Sample 5: 27.80 μm), suggesting that surface properties are influenced not only by the drying process but also by the uniformity of solvent exchange and freezing conditions. SEM investigations demonstrate that aerogels exhibit open, isotropic networks, whereas cryogels show localized densification. While scCO_2_ drying yields the most homogeneous and structurally intact aerogels, appropriately controlled lyophilization can provide comparable surface quality. Literature reports by Liebner et al. (2010)^31^, Illa et al. (2019)^32^, Vasconcellosa and Farinas (2018)^33^, and Machado et al. (2024)^34^ confirm that supercritical CO_2_ drying produces smoother, more homogeneous cellulose aerogels, while freeze-drying can increase surface roughness unless freezing conditions are well controlled.

**Fig. 6:**
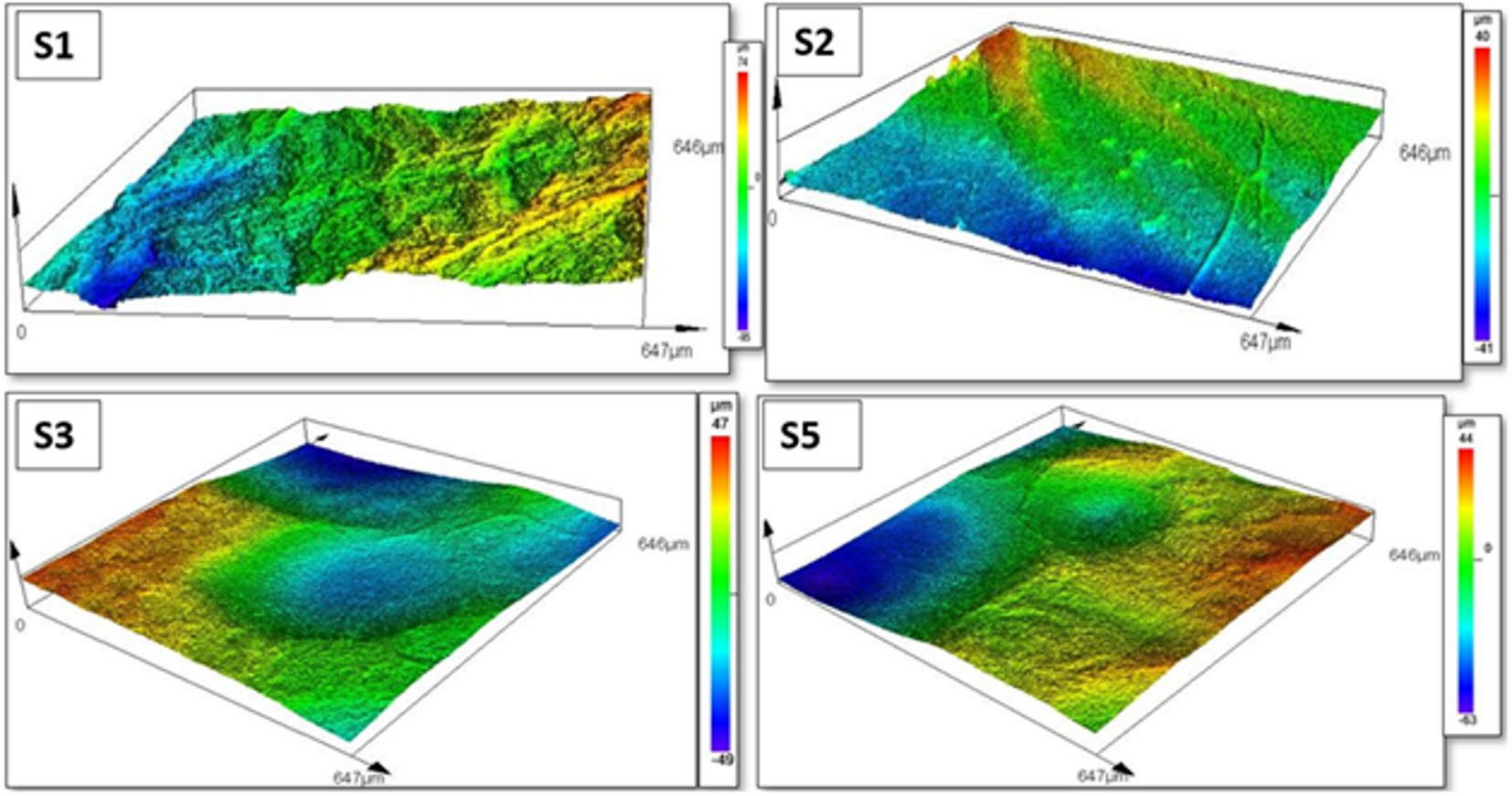
3D (3-dimensional) surface topography of BC samples.

However, three-dimensional (3D) confocal maps in Fig. 6 show significant differences in surface texture and height variation between aerogel and cryogel bacterial cellulose (BC) samples. These variations indicate the influence of drying methods on surface relief, mechanical relaxation, and nanofibrillar integrity.

Both aerogel samples exhibit well-preserved and continuous surface structures, although the surface height distributions differ significantly. Sample 1 shows pronounced surface undulations (height range = − 95 to + 74 μm), which may result from shrinkage or stress gradients during supercritical drying. However, SEM images confirm that the nanofibrillar network remains intact and highly porous.

Sample 2 exhibits a flatter and more homogeneous surface (height range ≈ − 41 to + 40 μm) with the lowest observed roughness (Rz = 21.49 μm), indicating effective solvent replacement, uniform depressurization, and minimal structural deformation. SEM micrographs of Sample 2 reveal uniformly distributed nanofibers and open pores, indicating a well-preserved aerogel structure resulting from controlled scCO_2_ drying.

In contrast, the cryogel samples (3 and 5) had slightly more compact and uneven topographies, which is consistent with the partial structural collapse shown in the SEM images. Sample 3 has moderate surface waviness (height range ≈ -49 to + 47 μm), with shallow depressions and dome-like elevations caused by uneven ice nucleation and incomplete sublimation. Sample 5 has slightly smoother but denser surface features (height range ≈ -63 to + 44 μm) due to fiber compaction and pore wall merging during drying. Sample 5 has a lower Rz (27.80 μm), although this is due to surface flattening caused by densification rather than greater microstructural preservation.

**Fig. 7:**
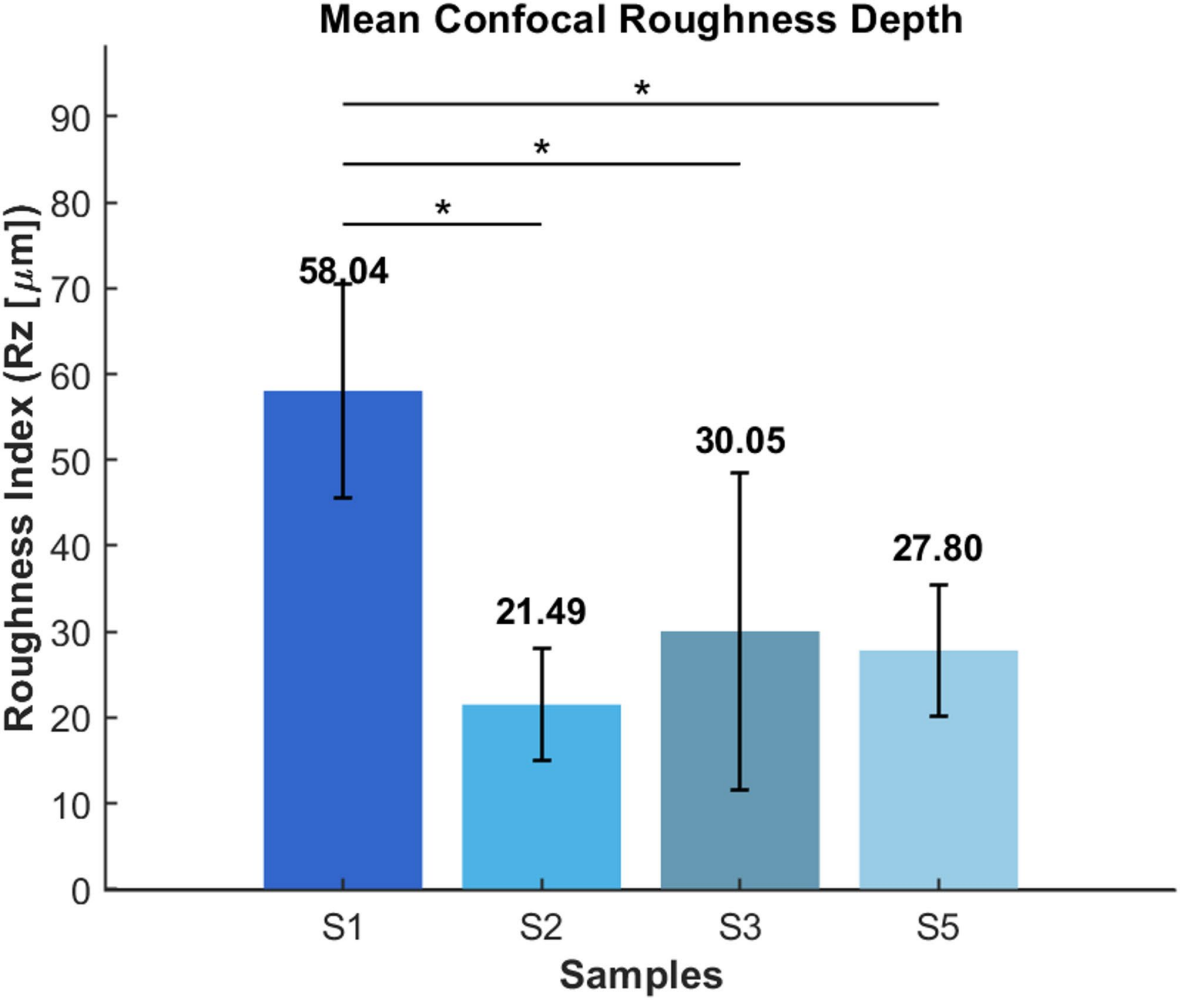
Mean confocal surface roughness (Rz [µm]) of BC aerogel & cryogel samples (*n* = 3). Bars represent mean ± SD (Standard deviation). Asterisks denote statistically significant differences between samples (*p* < 0.05, one-way ANOVA with Tukey’s post-hoc test).

As indicated in Table 2, the mean roughness index changed considerably between samples, as validated by one-way ANOVA (F(3, 8) = 5.28, *p* = 0.0267). The aerogel sample (S1) had the highest surface roughness (58.04 μm), while S2 (21.49 μm) and cryogel samples (S3 = 30.05 μm; S5 = 27.80 μm) had significantly lower values as shown in Fig. 7. The values of the cryogels S3 and S5 were in the middle, 30.05 μm and 27.80 μm, respectively. Tukey’s post-hoc (Tukey’s Honest Significant Difference) test revealed that S1 was substantially rougher than S2, S3, and S5 (*p* < 0.05), as seen in Table 3. There were no statistically significant differences between S2, S3, and S5. In this dataset, scCO_2_-dried aerogel (S2) produced the smoothest surface, whereas S1 had a rougher topology. Cryogels (S3, S5) were intermediate and statistically comparable to S2.

**Table 2:** One-way ANOVA results on BC samples.

ANOVA results					
Source	SS	df	MS	F	Prob > F
Groups	2363.8	3	787.9	5.28	0.00267
Error	1193.4	8	149.2		
Total	3557.2	11			

**Table 3:** Tukey post-hoc results on BC samples.

Tukey post-hoc results					
Group1	Group2	Lower	MeanDiff	Upper	*p* value
{‘S1’}	{‘S2’}	4.61	36.55	68.48	0.03
{‘S1’}	{‘S3’}	− 3.94	27.99	59.93	0.09
{‘S1’}	{‘S5’}	− 1.70	30.24	62.17	0.06
{‘S2’}	{‘S3’}	− 40.49	− 8.55	23.38	0.83
{‘S2’}	{‘S5’}	− 38.25	− 6.31	25.63	0.92
{‘S3’}	{‘S5’}	− 29.69	2.24	34.18	1.00

Although both were scCO_2_-dried, S1 had significantly higher surface roughness than S2, showing that differences in pre-drying processing (e.g., solvent exchange, gel composition/aging, or crosslinking) are the key drivers of the observed topography, rather than the drying method itself.

### **Specific** surface area and pore volume analysis/nitrogen sorption measurements

The BET analysis (Table 4) distinguishes the textural features of bacterial cellulose (BC) pellicles generated by supercritical CO_2_ drying and freeze-drying. After acetone solvent exchange and scCO_2_ drying, samples 1 and 2 exhibited nearly identical surface areas (123 and 122 m^2^/g), total pore volumes (0.36 and 0.35 cm^3^/g), and an average pore diameter of 10 nm. The mean BET surface area (123 m^2^/g) and total pore volume (0.36 cm^3^/g), together with a low standard deviation (0.8 m^2^/g and 0.01 cm^3^/g, respectively), indicate high reproducibility and homogeneous mesoporosity. The results confirm that solvent exchange successfully removed water and reduced capillary forces, allowing scCO_2_ drying to preserve the native nanofibrillar structure.

The large surface area and consistent pore diameter indicate a well-developed, open mesoporous network characteristic of true aerogels, which is consistent with SEM and confocal observations of interconnected fibrils and uniform porosity. These values are consistent with previously reported scCO_2_-dried BC aerogels (100–200 m^2^/g and 0.3–0.4 cm^3^/g), indicating that this drying method effectively maintains structural integrity and gas-accessible pore volume^31,32,34^. It should be noted, however, that N_2_ sorption analysis may underestimate the total pore volume, as macropores exceeding the accessibility range of condensed nitrogen are not detected by this technique.

**Table 4:** BET surface area, pore volume, and pore diameter results of BC aerogels and cryogels.

Sample	Drying method	BET surface area (m^2^/g)	Total pore volume (cm^3^/g)	Average pore diameter (nm)	Linear relative pressure range (*p*/*p*₀)	Degassing
S1	Supercritical CO_2_	124	0.36	9.8	0.05–0.3	50 °C, 48 + h
S2		122	0.35	9.8		
Mean		123	0.36	9.8		
Standard deviation		0.8	0.01	0		
S3	Freeze-dried	40	0.08	9.8		
S4		63	0.17	9.8		
S5		51	0.13	12.2		
Mean		51.3	0.13	10.6		
Standard deviation		11.4	0.04	1.4		

In contrast, the cryogels (Samples 3–5) were lyophilized with only 3 h of freezing during the drying process and without deep pre-freezing, resulting in significantly reduced surface area and pore volume, as well as increased variability. BET surface areas varied from 40 to 63 m^2^/g (mean 51.3 m^2^/g, σ = 11.4), with pore volumes ranging from 0.08 to 0.17 cm^3^/g (mean 0.13 cm^3^/g, σ = 0.04). Although the average pore diameter remained within the mesoporous range (10.6 nm), the increased standard deviation (1.4 nm) suggests greater structural heterogeneity. These findings indicate that the absence of regulated pre-freezing resulted in incomplete ice templating and uneven sublimation, which led to fibril aggregation, pore coalescence, and localized network collapse.

Cryogel samples exhibited significantly lower surface area and pore volume compared to aerogel samples, indicating a denser morphology, as observed in SEM and three-dimensional topography analyses. The data confirm that scCO_2_ drying with solvent exchange yields compositionally pure, highly porous, and structurally uniform BC aerogels, whereas lyophilization without deep pre-freezing results in compacted, less porous structures with diminished textural uniformity. These observations are consistent with previously reported trends in the drying behavior of nanocellulose-based aerogels.

**Fig. 8:**
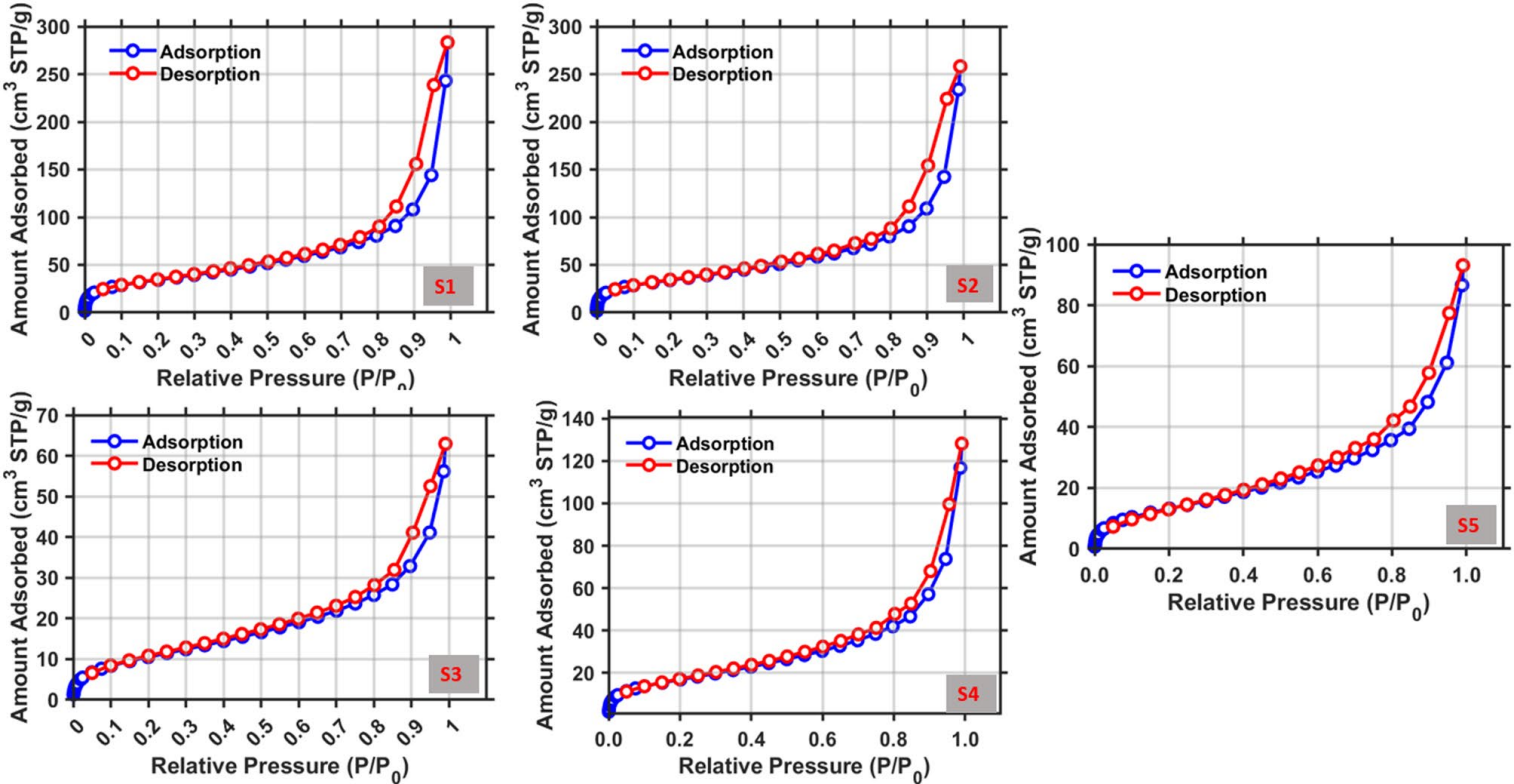
N_2_ adsorption–desorption isotherms of the BC samples.

N_2_ adsorption–desorption isotherms of the samples are shown in Fig. 8. All graphs confirm Type IV isotherms with H3 hysteresis loops (red adsorption, blue desorption), indicating mesoporous, slit-like pores formed between ribbon-like fibrils, with minimal true microporosity (very low uptake at p/p₀ < 0.05). Capillary condensation begins at p/p₀ = 0.8–0.9, and the hysteresis loop remains open at high p/p₀, which is characteristic of H3-type isotherms (plate-like aggregates with open macropores), as defined by the IUPAC classification (ISO 15901-2 / IUPAC, 2015)^35^. These trends are consistent with the BET results presented in Table 4, which show the accessible surface area and pore volume of scCO_2_-dried samples compared to lyophilized samples.

Sample 1 had the highest adsorbed amount: the red branch increases abruptly above p/p₀ ≈ 0.8, whereas the blue branch desorbs at lower uptake, forming a large but well-defined H3 loop. High S < sub> BET</sub> (~ 124 m^2^ g^−1^) and V < sub> p</sub> (~ 0.36 cm^3^ g^−1^) indicate abundant and homogeneous mesoporosity with stable pore walls.

Sample 2 exhibits a similar isotherm shape to Sample 1 but shows a slightly lower maximum uptake. The red and blue branches remain closer in the mid-p/p₀ range, resulting in a narrower loop. This indicates reproducible mesoporosity, with slightly reduced V < sub> p</sub> (S < sub> BET</sub > ~ 122 m^2^ g^−1^; V < sub> p</sub > ~ 0.35 cm^3^ g^−1^).

Sample 3 shows the lowest adsorption across the entire set, with a narrow H3 loop; the adsorption curve rises only marginally even at p/p₀ = 1. Table 2 shows that partial pore collapse and blocked pathways lead to the lowest S < sub> BET</sub> (≈ 40 m^2^ g^−1^) and V < sub> p</sub> (≈ 0.08 cm^3^ g^−1^) values. Sample 4 exhibits adsorption behavior intermediate between Samples 3 and 5; the adsorption curve peaks near p/p₀ ≈ 0.9, with a noticeable H3 loop. Table 2 shows S < sub> BET</sub > ≈ 63 m^2^ g^−1^ and V < sub> p</sub > ≈ 0.17 cm^3^ g^−1^, indicating moderate mesoporosity but lower uniformity than scCO_2_-dried samples.

Sample 5’s adsorption curve increases by more than threefold at high p/p₀. The loop is larger at saturation (the desorption branch lies substantially above the adsorption branch), indicating pore-neck bottlenecks (ink-bottle effects) and heterogeneous lamellar voids. This observation is consistent with Table 2 (S < sub> BET</sub > ≈ 51 m^2^ g^−1^; V < sub> p</sub > ≈ 0.13 cm^3^ g^−1^) and a slightly greater average pore diameter (12.16 nm), calculated from 4 V/A, attributed to pore coalescence.

scCO_2_ drying combined with acetone solvent exchange preserves an open and well-connected mesoporous network, characterized by high uptake and a regulated H3 loop, and is therefore suitable for acoustic absorption, thermal insulation, and filtration applications where accessible surface area and tortuous flow pathways are important. Among the solvents tested (ethanol, methanol, and acetone), acetone yielded the most stable specimens with minimal shrinkage and consistent results, confirming its suitability for further use. Lyophilized samples (3 h freezing) exhibit lower uptake and broader hysteresis loops, resulting in heterogeneous and partially densified networks. Such structures may be suitable for applications requiring stiffer or denser materials, such as composite fillers.

To facilitate direct comparison between the two drying methods, Table 5 summarizes the important structural parameters derived from SEM, BET, and confocal investigations.

**Table 5:** An overview of the structural and surface characteristics of BC aerogels and cryogels made using various drying techniques.

Parameter	ScCO_2_-dried BC aerogels	Lyophilized BC cryogels
Drying route	Acetone solvent exchange + supercritical CO_2_ drying	Direct lyophilization with integrated 3 h pre-freezing
Additives used	None	None
BET surface area (m^2^/g)	123	51.4
Total pore volume (cm^3^/g)	0.36	0.13
Average pore diameter (nm)	10	10.6
Theoretical porosity (mean, %)	99.1	98.8
Surface roughness, Rz (µm)	S1: 58.04; S2: 21.49	S3: 30.05; S5: 27.80
SEM nanofibrillar morphology	Highly interconnected, uniform nanofibrillar network with smooth and well-dispersed fibrils	Preserved nanofibrillar framework with localized fibril aggregation and densification
Pore structure (SEM/confocal)	Homogeneous, open, and isotropic pore distribution	More heterogeneous pore distribution with partial structural compaction
Chemical composition (EDS)	High-purity cellulose; dominant C and O signals	Minor Ca, Na, and Si traces originating from the culture medium
FTIR features	Characteristic cellulose I bands preserved	Cellulose I bands preserved; enhanced O–H stretching intensity
Structural reproducibility	Higher structural uniformity observed across samples by SEM and confocal microscopy	Greater variability and heterogeneity observed by SEM and confocal microscopy

## Conclusions

The extensive structural, morphological, and textural investigations demonstrate that the drying technique critically determines the quality and functional utility of bacterial cellulose (BC) pellicles. Acetone solvent exchange followed by supercritical CO_2_ (scCO_2_) drying yielded improved physicochemical and morphological outcomes compared to direct lyophilization with limited freezing. scCO_2_-dried aerogels exhibited high BET surface areas (~ 123 m^2^/g), total mesopore volumes (~ 0.36 cm^3^/g), and uniform mesopore sizes (~ 10 nm), indicating enhanced preservation of the three-dimensional nanofibrillar network and improved structural homogeneity. Nitrogen physisorption, which probes the mesopore range (2–50 nm), together with SEM and confocal microscopy, confirms the presence of a hierarchically porous architecture combining meso- and macroporosity.

In contrast, cryogel samples exhibited lower mean surface area (51.4 m^2^/g) and pore volume (0.13 cm^3^/g), along with greater variability and partial structural densification. The brief three-hour freezing step during lyophilization provided limited ice templating, resulting in heterogeneous pore structures, localized fiber compaction, and reduced surface roughness. Surface roughness analysis revealed significant differences among samples (one-way ANOVA: F(3, 8) = 5.28, *p* = 0.0267), with the scCO_2_-dried aerogel S1 exhibiting the highest roughness (58.04 μm), while S2 (21.49 μm) and cryogels S3 (30.05 μm) and S5 (27.80 μm) showed lower values. Tukey’s post-hoc test confirmed that S1 was significantly rougher than S2, S3, and S5 (*p* < 0.05), whereas no significant differences were observed among the latter group. The marked roughness difference between S1 and S2, despite identical scCO_2_ drying, underscores the dominant role of pre-drying processing history in governing surface topography rather than the drying method alone.

FTIR spectra of all samples retained the characteristic cellulose I bands, confirming preservation of the native molecular structure under both drying regimes. Subtle intensity variations, particularly enhanced O–H stretching in lyophilized samples, suggest increased bound moisture and modified hydrogen bonding consistent with microstructural densification. Combined SEM, EDS, confocal microscopy, and BET analyses therefore provide a comprehensive and internally consistent picture of structure–processing relationships achieved without chemical additives or post-synthetic modification.

Overall, solvent-exchanged scCO_2_ drying produces BC aerogels with highly porous, reproducible mesostructures and superior structural uniformity. Importantly, the results also demonstrate that direct, additive-free lyophilization yields cryogels with largely preserved nanofibrillar architecture and comparable bulk porosity, albeit with increased heterogeneity. This finding represents the central contribution of this work: the identification of a performance–sustainability trade-off between two simple and scalable drying routes, rather than a binary superiority of one method over the other.

While scCO_2_-dried aerogels are preferable for applications requiring precise structural control and high reproducibility, lyophilized cryogels offer a cost-effective, operationally simple, and environmentally benign alternative when minor pore heterogeneity is acceptable. By quantitatively comparing structural preservation using multiple complementary techniques, this study provides a practical framework for selecting BC drying strategies based on targeted functional requirements, sustainability considerations, and processing simplicity.

In order to further improve the pore homogeneity and surface morphology of BC cryogels, future research may concentrate on enhancing additive-free lyophilization conditions, such as freezing time and temperature. Furthermore, making a connection between the observed structural characteristics and application-related performance (such as acoustic and thermal insulation) can help choose drying techniques for sustainable and scalable BC-based materials.

## Data Availability

The original contributions presented in this study are included in the article. Further inquiries can be directed to the corresponding authors.
